# Integrated fecal microbiota and metabolomics analysis of the orlistat intervention effect on polycystic ovary syndrome rats induced by letrozole combined with a high-fat diet

**DOI:** 10.1186/s13048-023-01193-3

**Published:** 2023-06-05

**Authors:** Jianmei Yang, Enli Wang, Mingmin Jiang, Yujun Tan, Fangfang Yao, Chenghong Sun, Lihong Pan, Ling Gao, Jingchun Yao

**Affiliations:** 1 Department of Pediatric Endocrinology, Shandong Provincial Hospital, Shandong University, Jinan, 250021 Shandong China; 2 State Key Laboratory of Integration and Innovation of Classic Formula and Modern Chinese Medicine, Lunan Pharmaceutical Group Co. Ltd., Linyi, 276006 Shandong China; 3 Department of Pediatric Endocrinology, Shandong Provincial Hospital affiliated to Shandong First Medical University, Jinan, 250021 Shandong China; 4 Scientific Center, Shandong Provincial Hospital, Shandong University, Jinan, 250021 Shandong China

**Keywords:** Orlistat, Obesity, Polycystic ovary syndrome, Gut microbiota, Metabolomics

## Abstract

**Background:**

This study aimed to compare the characteristics of the gut microbiota and their metabolite profiles between polycystic ovary syndrome (PCOS) and orlistat-treated PCOS rats (ORL-PCOS), which could help to better understand the underlying mechanism of the effect of orlistat on PCOS.

**Methods:**

PCOS rat models were established using letrozole combined with a high-fat diet. Ten rats were randomly selected as a PCOS control group (PCOS). The other three groups (*n* = 10/group) were additionally supplemented with different doses of orlistat (low, medium, high). Then, fecal samples of the PCOS and ORL-PCOS groups were analysed by 16S rRNA gene sequencing and untargeted metabolomics. Blood samples were collected to detect serum sex hormones and lipids.

**Results:**

The results showed that orlistat attenuated the body weight gain, decreased the levels of T, LH, the LH/FSH ratio, TC, TG and LDL-C; increased the level of E2; and improved estrous cycle disorder in PCOS rats. The bacterial richness and diversity of the gut microbiota in the ORL-PCOS group were higher than those in the PCOS group. The ratio of *Firmicutes* to *Bacteroidetes* was decreased with orlistat treatment. Moreover, orlistat treatment led to a significant decrease in the relative abundance of *Ruminococcaceae* and *Lactobacillaceae*, and increases in the abundances of *Muribaculaceae* and *Bacteroidaceae*. Metabolic analysis identified 216 differential fecal metabolites in total and 6 enriched KEGG pathways between the two groups, including steroid hormone biosynthesis, neuroactive ligand-receptor interaction and vitamin digestion and absorption. Steroid hormone biosynthesis was the pathway with the most significant enrichment. The correlations between the gut microbiota and differential metabolites were calculated, which may provide a basis for understanding the composition and function of microbial communities.

**Conclusions:**

Our data suggested that orlistat exerts a PCOS treatment effect, which may be mediated by modifying the structure and composition of the gut microbiota, as well as the metabolite profiles of PCOS rats.

**Supplementary Information:**

The online version contains supplementary material available at 10.1186/s13048-023-01193-3.

## Introduction

Polycystic ovary syndrome (PCOS), a complex gynaecological endocrine and metabolic disorder, is characterized by polycystic ovarian morphology (PCOM), infrequent ovulation or anovulation, clinical or biochemical hyperandrogenism, and at least 2 of 3 features are needed to establish a diagnosis [1, 2]. PCOS is also associated with several metabolic disturbances, such as insulin resistance (IR), increased risk of type 2 diabetes mellitus (T2DM) and obesity [1, 3]. The prevalence of PCOS is between 8 and 13% according to the population studied and the definitions used [4].

Studies have found that metabolic factors play important roles in PCOS pathological mechanisms. Some PCOS related genes were found to be related to carbohydrate metabolism and steroid synthesis pathway [5]. Metabolic imbalance could lead to PCOS development [6]. Several studies have shown that PCOS patients have abnormal metabolite composition, including bile acids, short-chain fatty acids and branched-chain amino acids [7, 8]. In addition, gut microbiota (GM) dysbiosis was also found in PCOS development. The GM plays a key role in human and animal health, and maintains a dynamic balance to prevent the development of various diseases. In recent years, evidence has been provided on the correlation between the GM and the development of metabolic diseases, such as obesity and T2DM [9], which accordingly leads to the hypothesis that the GM is closely associated with the aetiology and pathological mechanisms of PCOS [6, 10, 11]. Many studies have investigated this relationship. For example, compared with healthy women, PCOS patients had lower α diversity of the GM, which correlated with the increase in androgens [12]. Sun et al. found that plasma dimethylamine, a product of choline metabolism by the GM, was significantly increased in PCOS patients, demonstrating that GM growth was increased in the group of women with PCOS [13]. In addition, it seems that abnormal short-chain fatty acid (SCFA) metabolism caused by an abnormal GM is associated with IR and hyperandrogenaemia in PCOS patients [14]. These studies have confirmed the close relationship between the GM, metabolites and PCOS.

Over 50% of PCOS cases are overweight [15]. Evidence implies that obesity worsens some features of PCOS, such as infertility, hyperandrogenaemia and IR [16–18]. Obesity prevention and treatment will benefit patients with PCOS [18]. Weight loss is the first-line treatment for overweight women with PCOS [19]. Orlistat, an anti-obesity drug approved by the U.S. FDA, has been used to improve or reverse the pathological characteristics of PCOS [20]. Kumar et al. found that orlistat in PCOS is as effective as metformin in reducing weight with improvement in the lipid profile and pregnancy rates [21]. Orlistat could also combine with a low-calorie diet to improve IR and reduce circulating androgens [22]. There is relatively little research on the mechanism by which orlistat improves PCOS. Considering the relationship between the GM, metabolites and PCOS, in the current study, we therefore aimed to compare the characteristics of the gut microbiota and their metabolite profiles between the PCOS and ORL-PCOS groups, which could help to better understand the mechanism of the effect of orlistat on PCOS.

## Materials and methods

This study was performed in accordance with the ethical standards laid down in the Declaration of Helsinki, and the Medical Ethics Committee of Lunan Pharmaceutical Group Co., Ltd approved all procedures (Permit No. AN-IACUC-2021–067).

### Animals

**Fig. 1:**
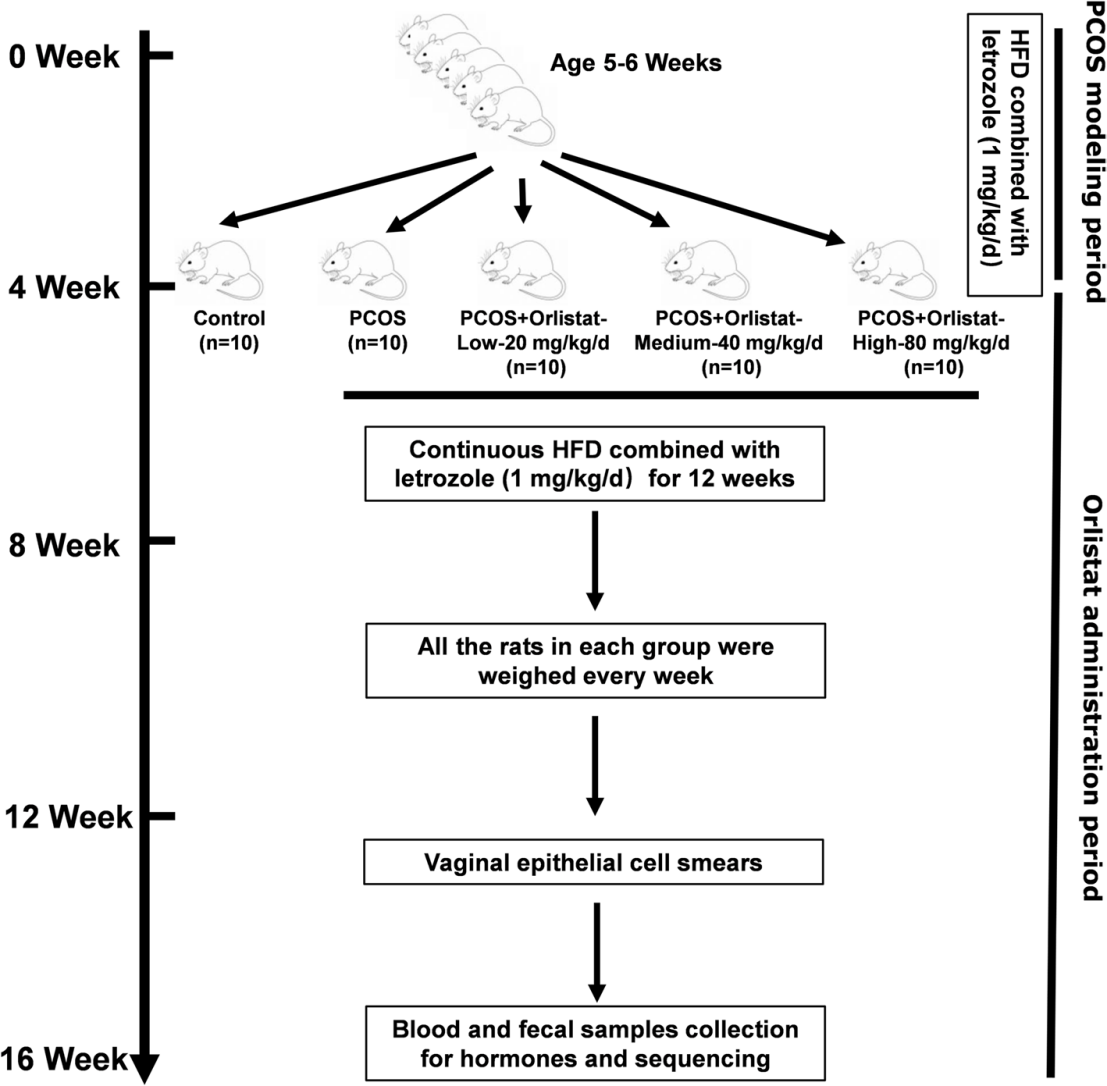
Schematic diagram of the animal experiment design and timeline

Specific pathogen-free female rats (5–6 weeks, 145–165 g, Animal Qualification Certificate No.370726211100731686) were purchased from Beijing Vital River Laboratory Animal Technology Co., Ltd. (Certificate No. SCXK, 2016–0006; Beijing, China). Before the experiment, all the rats were fed adaptively and quarantined for 1 week. Rats were housed in a breeding room under standard conditions (20–26 °C, 40–70% relative humidity, centralized ventilation of central air conditioner ≥ 15 times per hour, 12-h dark/light cycle). During the experiment, ten rats were randomly selected as a control group (control) and were fed a high-fat diet with 45% of kcal from fat (OpenSource Diets™ Research Diets #D12451). The other rats had free access to water and were fed a high-fat diet (HFD) with 45% of kcal from fat combined with letrozole (1 mg/kg/d. Jiangsu Hengrui Pharmaceutical Co., Ltd; Jiangsu, China) for 28 days to generate a PCOS model [23]. Forty successfully letrozole-induced PCOS rats were randomly selected and divided into four groups (*n* = 10/group). Ten rats were randomly selected as a PCOS control group (PCOS). The remaining thirty rats were randomly allocated into three groups, which were then supplemented with different doses of orlistat (low-20 mg/kg/d; medium-40 mg/kg/d; high-80 mg/kg/d; Lunan Pharmaceutical Group Corporation; Shandong, Linyi, China.) over the next 12 weeks. The dose of orlistat was selected based on a previous study [24]. Letrozole was given throughout the experiment (12 weeks) to the forty PCOS rats. The control group and PCOS group were given the same volume of solvent without orlistat. Orlistat or solvent was administered by oral gavage. Vaginal epithelial cell smears were taken daily for 10 consecutive days to analyse the estrous-cycle stage. Schematic diagram of the animal experiment design and timeline is listed in Fig. 1.

### Sample collection

After orlistat treatment, the rats were weighed. At the end of the experiment, Lee’s index was calculated as previously described [25]. Rats in each group fasted for 24 h after the last administration. Blood was taken from the abdominal aorta after sacrificing the rat. Blood samples were collected and then centrifuged at 4,000 rpm for 10 min at 4 °C. The levels of serum sex hormones including testosterone (T), oestradiol (E2), follicle stimulating hormone (FSH) and luteinizing hormone (LH) were tested using radioimmunoassay (Beijing North Institute of Biotechnology Co., Ltd. Beijing, China) according to the specifications for each kit. Biochemical assays were used to analyse lipids, including total cholesterol (TC), triglycerides (TGs), high- density lipoprotein cholesterol (HDL-C) and low-density lipoprotein cholesterol (LDL-C). Data were analysed with SPSS 18.0 (IBM SPSS, Armonk, NY, USA) using the Mann–Whitney U test and expressed as the mean ± standard deviation (SD). A *p* value of < 0.05 was considered statistically significant. Fresh fecal samples (1–3 g) of the PCOS group and the high-doses orlistat treatment PCOS group (ORL-PCOS) were collected in sterile plastic tubes, and then stored at -80 °C for subsequent analyses.

### DNA extraction and 16S rRNA gene sequencing

Total bacterial DNA was extracted using the CTAB/SDS method. DNA concentration and purity were monitored on 1% agarose gels. DNA was then diluted to 1 ng/μl using sterile water. 16S rRNA genes were amplified by PCR (98 °C for 1 min, followed by 30 cycles of denaturation at 98 °C for 10 s, annealing at 50 °C for 30 s, elongation at 72 °C for 60 s, and 72 °C for 5 min) using the specific primer (16S V3-V4: 341F-806R). All PCRs were carried out in 30 μL reactions with 15 μL of Phusion® High-Fidelity PCR Master Mix (New England Biolabs); 0.2 μM of forward and reverse primers, and approximately 10 ng of template DNA. PCR products were mixed with the same volume of 1X loading buffer containing SYBR Green), and electrophoresis was performed on a 2% agarose gel for detection. Samples with bright main bands between 400–450 bp were chosen for further experiments. PCR products were mixed in equidensity ratios. Then, the mixed PCR products were purified with an AxyPrepDNA Gel Extraction Kit (AXYGEN). Sequencing libraries were generated using the NEB Next®Ultra™DNA Library Prep Kit for Illumina (NEB, USA) following the manufacturer’s recommendations. The library was sequenced on an Illumina MiSeq/HiSeq2500 platform and 250 bp/300 bp paired-end reads were generated.

### Analysis of the gut microbiota

Paired-end reads from the original DNA fragments were merged using FLASH and assigned to each sample according to the unique barcodes. Sequence analysis was performed by the UPARSE software package using the UPARSE-OTU and UPARSE-OTUref algorithms. Sequences with ≥ 97% similarity were assigned to the same OTUs. In-house Perl scripts were used to analyse alpha diversity. Two metrics were calculated: the Chao1 and Shannon indices. For beta diversity analysis, principal coordinate analysis (PCoA) between the two groups based on both weighted and unweighted UniFrac algorithms was calculated using QIIME. The linear discriminant analysis (LDA)-effect size (LEfSe) method was used for the quantitative analysis of biomarkers within different groups. An LDA threshold > 3 was used as a threshold to identify the most differentially abundant taxa. Correlations between fecal microbiota and metabolites were analysed with R language (R 3.4.2).

### Metabolite extraction and LC–MS/MS analysis

Metabolites in the stool samples (60 mg) were extracted as previously described [26]. Briefly, sample metabolites were extracted using 400 μL of a methanol: water (4:1, v/v) solution. Then, the mixture was treated by a high-throughput tissue crusher at -20 °C, and 50 Hz for 6 min, vortex mixed for 30 s, ultrasonicated at 40 kHz for 30 min at 5 °C, and placed at -20 °C for 30 min to precipitate proteins. The mixture was centrifuged for 15 min (13,000 g, 4 °C). The supernatant was dried in a vacuum centrifuge. For LC–MS analysis, the samples were redissolved in 100 μL of acetonitrile/water (1:1, v/v) solvent. Analysis was performed using an UHPLC (1290 Infinity LC, Agilent Technologies) coupled to a quadrupole time-of-flight (AB Sciex TripleTOF 6600) at Shanghai Applied Protein Technology Co., Ltd. For HILIC separation, samples were analysed using a 2.1 mm × 100 mm ACQUITY UPLC BEH 1.7 µm column (Waters, Ireland). In both ESI positive and negative modes, the mobile phase contained A = 25 mM ammonium acetate and 25 mM ammonium hydroxide in water and B = acetonitrile. The gradient was 85% B for 1 min, linearly reduced to 65% in 11 min, reduced to 40% in 0.1 min, maintained for 4 min, and then increased to 85% in 0.1 min, with a 5 min re-equilibration period employed.

### Metabolomics analysis

The raw MS data were converted to MzXML files using ProteoWizard MSConvert before importing into freely available XCMS software. After normalization to the total peak intensity, the processed data were analysed by the R package, where they were subjected to multivariate data analysis, including orthogonal partial least-squares discriminant analysis (OPLS-DA). The sevenfold cross-validation and response permutation testing were used to evaluate the robustness of the model. The variable importance in the projection (VIP) value of each variable in the OPLS-DA model was calculated to indicate its contribution to the classification. Metabolites with VIP values > 1 were further subjected to Student’s t-test at the univariate level to measure the significance of each metabolite, and a *p* value of < 0.05 was considered statistically significant. For pathway enrichment analysis, the differential metabolites were annotated using the Kyoto Encyclopedia of Genes and Genomes (KEGG) database (https://www.kegg.jp/kegg/pathway.html).

### Statistical analysis

Values are expressed as the mean ± standard deviation (SD). All data were analyzed using SPSS 18.0 (International Business Machines Corporation, Armonk, New York, USA). The body weight, Lee’s index, serum sex hormones, and lipid profiles were analyzed by one-factor analysis of variance (ANOVA), followed by post-hoc the least significance difference (LSD)-t test. *p* < 0.05 was deemed statistically significant in all tests.

## Results

### Effects of orlistat on weight, sex hormones levels, lipids and the estrous cycle

Compared with the control (CON) group, the weight of the PCOS rats was significantly higher from week 4 to week 16. Meanwhile, the Lee’s index of the PCOS group was also significantly increased (Table 1). The weight and Lee’s indices of the rats in all the orlistat treatment groups were significantly decreased (*p* < 0.05) compared with those of the PCOS group.

**Table 1:** Effects of Orlistat on body weight and Lee’s index in PCOS rats

Group	Body mass					Lee’s index
	0 weeks	4 weeks	8 weeks	12 weeks	16 weeks	
Control	180.09 ± 8.75	251.13 ± 19.44^**^	312.54 ± 30.11^**^	309.46 ± 27.58^**^	349.53 ± 26.97^**^	3.08 ± 0.16^*^
PCOS	180.54 ± 7.81	316.60 ± 16.18	406.70 ± 25.18	435.97 ± 34.00	466.04 ± 32.81	3.21 ± 0.10
Low- Orlistat	179.97 ± 8.60	307.84 ± 28.22	379.21 ± 19.58^*^	408.28 ± 22.28^*^	433.35 ± 20.90^*^	3.11 ± 0.09^*^
Medium- Orlistat	179.83 ± 7.36	308.44 ± 22.08	375.92 ± 28.66^*^	405.68 ± 33.08^*^	425.75 ± 31.12^**^	3.09 ± 0.12^*^
High-Orlistat	180.29 ± 7.94	303.44 ± 18.09	372.59 ± 28.44^**^	390.93 ± 30.21^**^	409.00 ± 30.54^**^	3.08 ± 0.06^*^

Values are the mean ± SD (*n* = 10/group)

0 weeks: PCOS modeling start time; 4 weeks: established PCOS model and start time point of Orlistat administration; 8 weeks: 4 weeks after Orlistat administration, and so on

^*^p < 0.05

^**^p < 0.01 compared with the PCOS group

Compared with those in the CON group, the levels of serum T, and LH, and the ratio of LH/FSH were significantly higher (*p* < 0.05), and E2 was significantly lower (*p* < 0.01) in the PCOS group (Table 2). The rats in the high-orlistat group had significantly lower levels of T, LH, and the ratio of LH/FSH, and higher levels of E2 compared with those of the PCOS group. In addition, compared with the CON group, the PCOS rats had significantly higher concentrations of TC, TG and LDL-C (Table 2). The rats in the high- and medium-orlistat group had significantly lower levels of TC, TG and LDL-C than those in the PCOS group. No differences in HDL-C levels were found.

**Table 2:** Effect of supplementation with orlistat on serum sex hormone and lipids levels in PCOS rats

Group	Control	PCOS	Low- Orlistat	Medium- Orlistat	High-Orlistat
T (ng/ml)	1.51 ± 0.68^*^	4.83 ± 2.89	4.64 ± 2.49	3.10 ± 2.73	0.94 ± 0.72^*^
E2 (pg/ml)	14.40 ± 1.59^**^	2.47 ± 0.65	2.60 ± 0.84	2.99 ± 0.74	5.13 ± 0.29^**^
LH (mIU/ml)	5.18 ± 1.05^*^	7.03 ± 1.47	5.47 ± 1.06^*^	4.93 ± 0.93^**^	4.70 ± 1.49^**^
FSH (mIU/ml)	1.77 ± 0.32	1.71 ± 0.20	1.73 ± 0.31	1.76 ± 0.14	1.81 ± 0.46
LH/FSH	2.95 ± 0.29^*^	4.17 ± 1.19	3.33 ± 1.15	2.82 ± 0.66^*^	2.64 ± 0.74^*^
TC (mmol/L)	1.63 ± 0.29^*^	2.22 ± 0.50	1.72 ± 0.35	1.41 ± 0.48^**^	1.31 ± 0.22^**^
TG (mmol/L)	0.63 ± 0.11^**^	1.19 ± 0.34	0.84 ± 0.21^*^	0.70 ± 0.23^**^	0.65 ± 0.17^**^
LDL-C (mmol/L)	0.25 ± 0.03^*^	0.34 ± 0.06	0.25 ± 0.05^*^	0.24 ± 0.08^**^	0.21 ± 0.06^**^
HDL-C(mmol/L)	1.42 ± 0.16	1.30 ± 0.30	1.19 ± 0.23	1.02 ± 0.29	1.04 ± 0.19

Values are the mean ± SD (*N* = 5–7). Data were analyzed by one-factor analysis of variance (ANOVA), followed by post-hoc the least significance difference (LSD)-t test

*Abbreviation*: *T* Testosterone, *E2* Estradiol, *FSH* Follicle stimulating hormone, *LH* luteinizing hormone, *TC* Total cholesterol, *TG* Triglycerides, *HDL-C* High density lipoprotein cholesterol, *LDL-C* Low density lipoprotein cholesterol

^*^p < 0.05

^**^p < 0.01 compared with the PCOS group

**Fig. 2:**
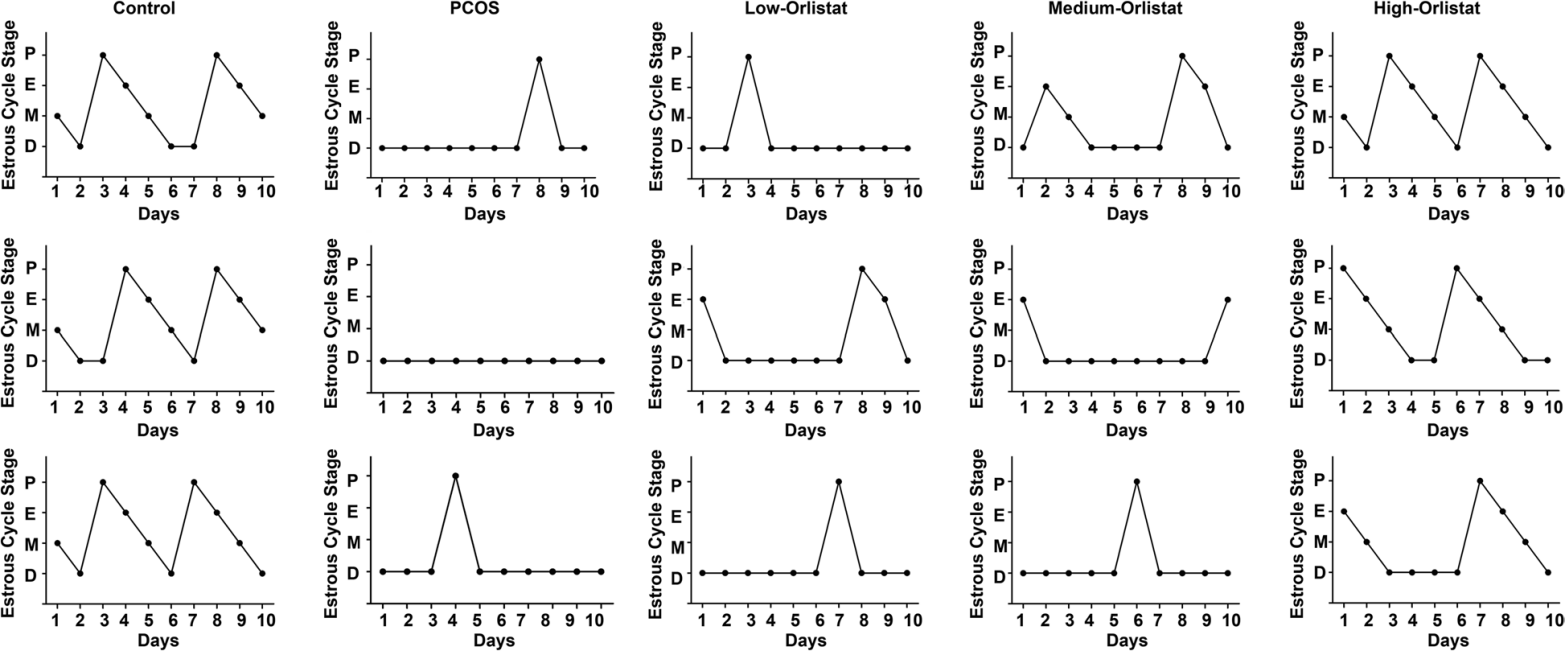
Effects of orlistat treatment on the estrous cycle. Three rats were randomly selected in each group and representative estrous cycles of the different groups are shown. P: Proestrus, E: Estrous, M: Metoestrus, D: Dioestrus

Estrous cycle assessment revealed that PCOS rats experienced a longer dioestrus and shorter proestrus and estrous than control rats (Fig. 2). Following orlistat treatment, the estrous cycle disorder was restored. The improvement effect on the estrous cycle disorder of the high-orlistat group was better than that of the low-orlistat group and medium-orlistat group.

### Effects of orlistat on the gut microbiota in PCOS mice

**Fig. 3:**
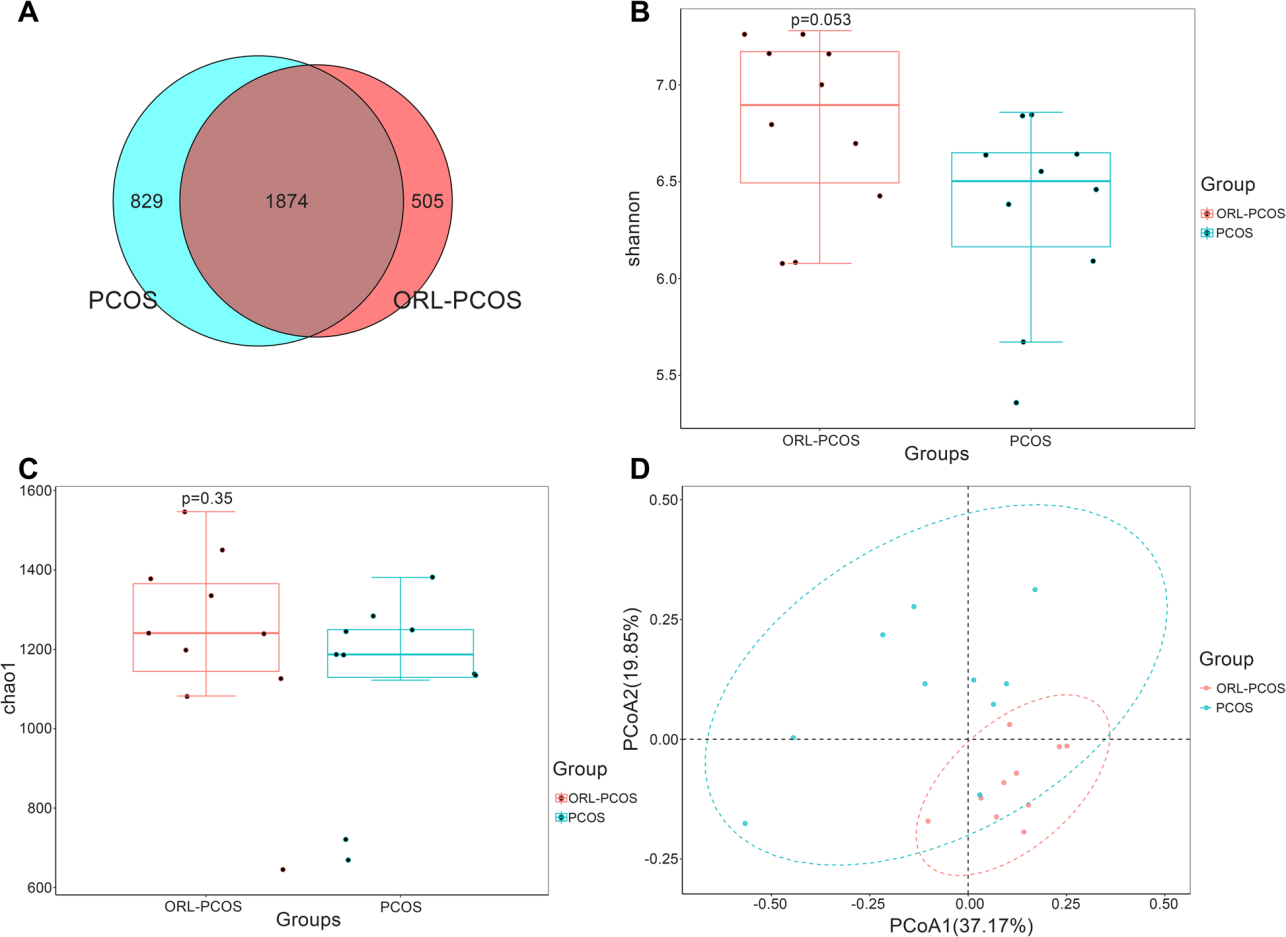
Characteristics of the gut microbiota after orlistat treatment. **A** Venn diagram of OTUs. The overlapping section represents the shared OTUs. There was no significant difference in the Shannon index. **B** and Chao1 (**C**) index between the ORL-PCOS and PCOS groups. **D** PCoA of the gut microbiota of ORL-PCOS and PCOS group based on weighted UniFrac algorithms

**Fig. 4:**
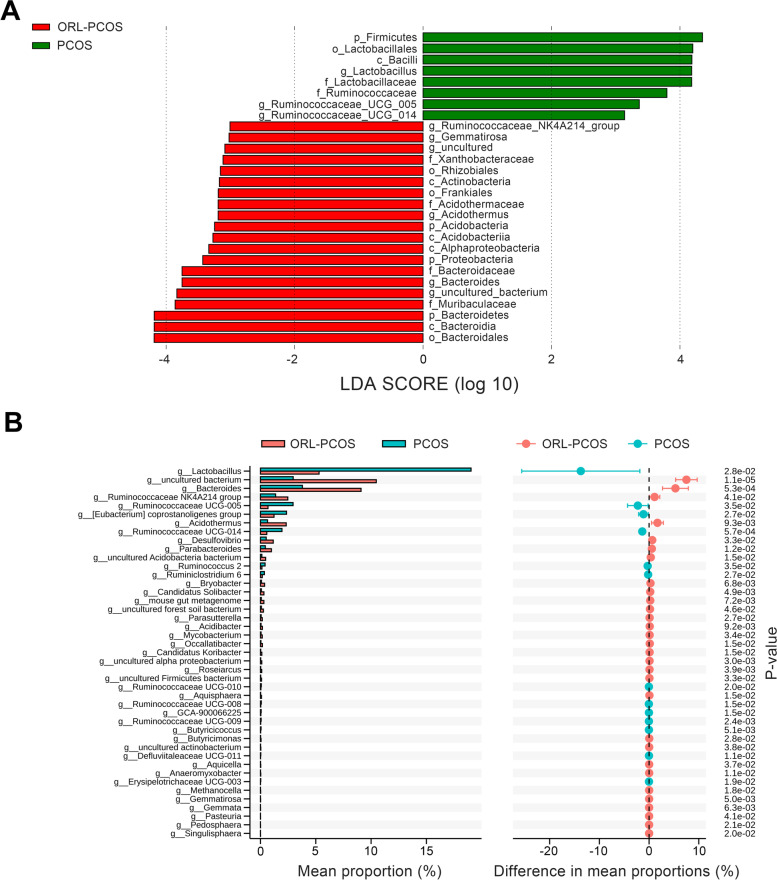
LEfSe of the gut microbiota of the ORL-PCOS and PCOS groups. **A** LEfSe taxonomic cladogram. **B** LDA chart (p = phylum, c = class, o = order, f = family, g = genus). LDA scores higher than 3.0 were used as a threshold for significance in LEfSe analyses

A total of 3,208 OTUs were obtained in this study (Fig. 3A). Then the Shannon index and Chao1 index (Fig. 3B and C) were calculated. No significant differences were observed between the diversity of the gut microbiota in the ORL-PCOS and PCOS groups. PCoA between groups based on the weighted UniFrac (Fig. 3D) algorithms was performed and showed a separation between the samples in these two groups. LEfSe (Fig. 4A and B) was used to analyse biomarkers in the microbiota of each group. The PCOS group was significantly enriched in *Lactobacillus, Ruminococcaceae_UCG_005* and *Ruminococcaceae_UCG_014*. The ORL-PCOS group was significantly enriched in *Bacteroides* and *Muribaculaceae*.

### Effects of orlistat on fecal metabolomics in PCOS mice

**Fig. 5:**
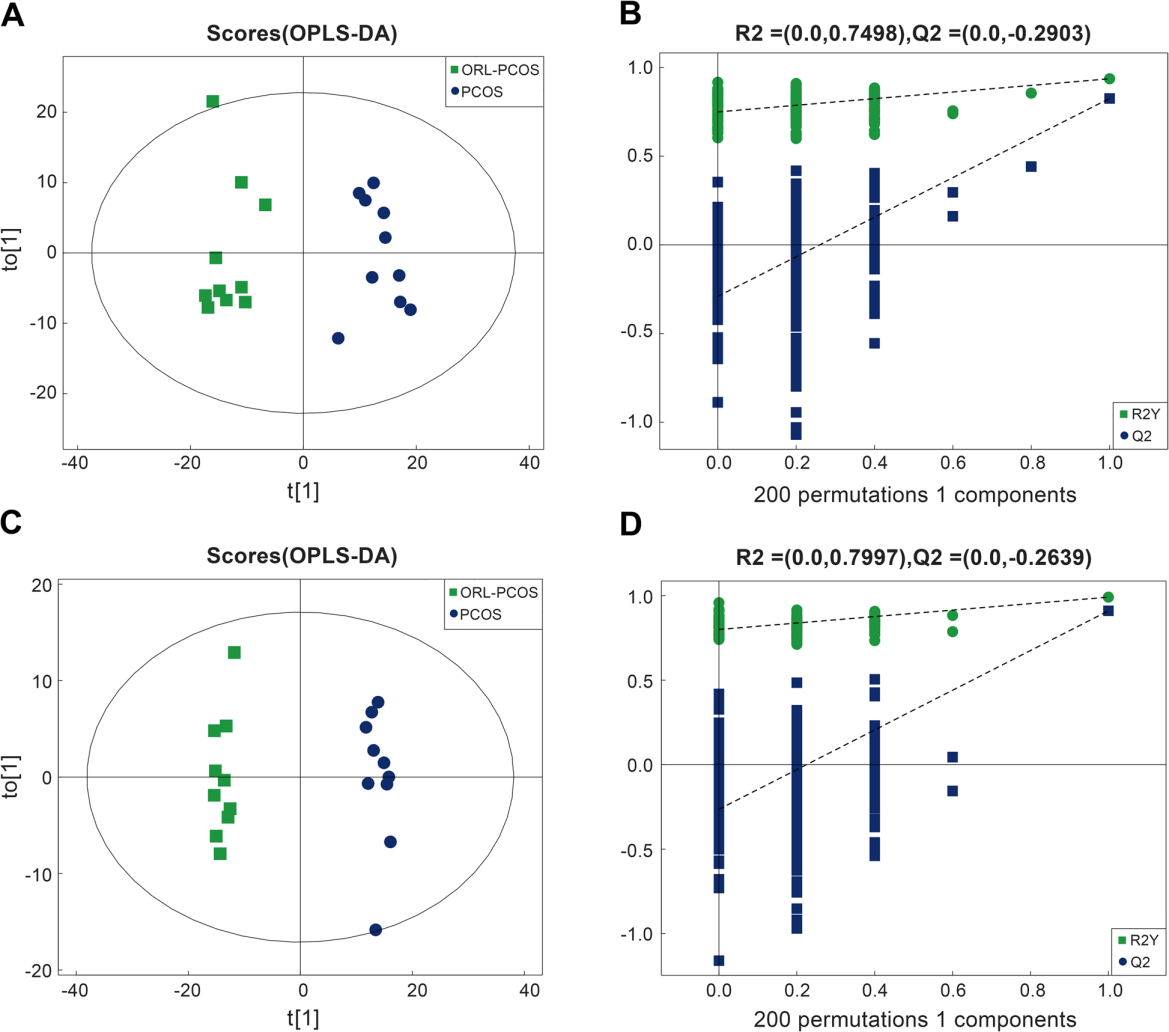
Multivariate statistical analyses of fecal metabolites in the ORL-PCOS and PCOS groups. **A** OPLS-DA negative ion mode scatter diagram. **B** Corresponding validation plots of the OPLS-DA negative ion mode obtained from the permutation test. **C** OPLS-DA positive ion mode scatter diagram. **D** Corresponding validation plots of the OPLS-DA positive ion mode obtained from the permutation test

**Fig. 6:**
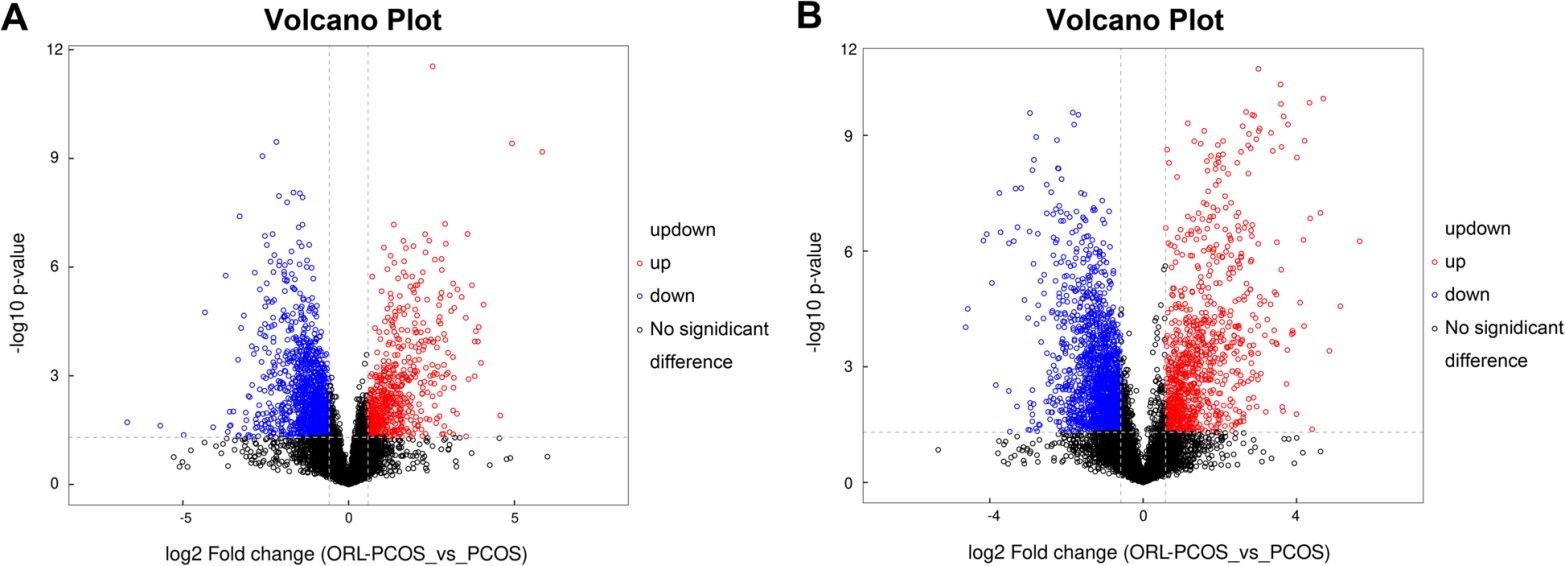
Volcanic map analysis in negative (**A**) and positive (**B**) ion modes. According to the VIP value, *p* value and FC value, volcano maps were drawn to display the overall distribution of the differential metabolites. The blue dots represent significantly downregulated metabolites in the ORL-PCOS group, and the red dots represent significantly upregulated differential metabolites. The black dots represent nonsignificant differential metabolites

**Fig. 7:**
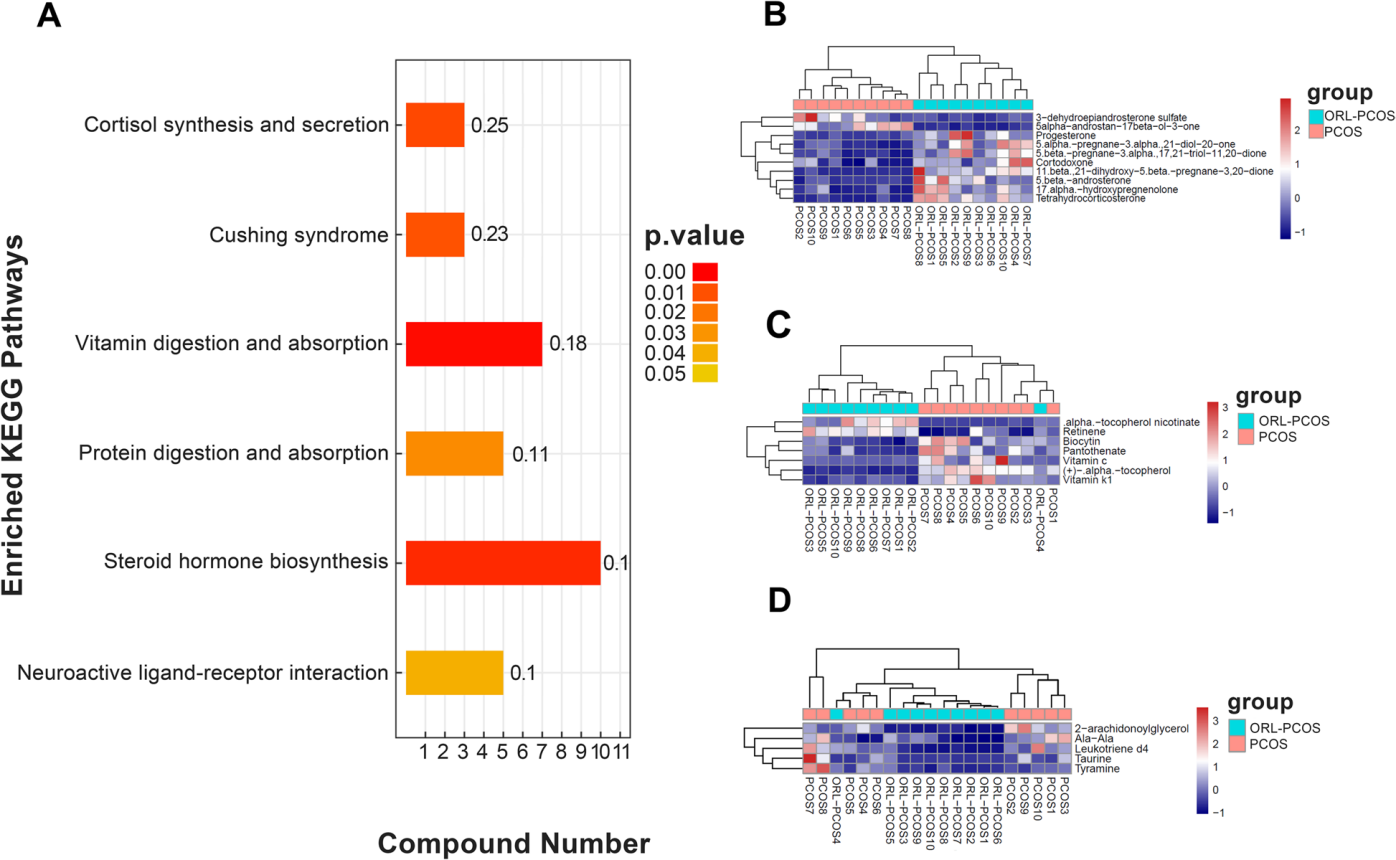
KEGG pathway enrichment analysis of differential metabolites. **A** The ordinate represents the name of the pathway, and the abscissa represents the number of differentially expressed metabolites included in each KEGG metabolic pathway. The colour indicates the *p* value of enrichment analysis. The number on the column represents the rich factor. **B** Heatmap for steroid hormone biosynthesis, **C** Heatmap for vitamin digestion and absorption, **D** Heatmap for neuroactive ligand-receptor interaction

OPLS-DA was performed to verify differential the metabolites between the two groups (Fig. 5A and C), and was validated by permutation analysis (Fig. 5B and D). The results showed clear separation between the ORL-PCOS and PCOS groups, suggesting a significant difference in fecal metabolites. In this study, VIP > 1.0 was used to screen metabolite differences, and only metabolites identified with a fold change (FC) > 1.5 or < 1/1.5, *p* < 0.05 and VIP > 1.0 were considered differential. Based on the FC and *p* values, volcanic diagrams were made to illustrate the up- and down-regulated metabolites between the two groups (Fig. 6A and B). All detailed information on the different metabolites is listed in Supplementary Table 1. In addition, the differential metabolites were enriched and analysed based on the KEGG database (Fig. 7A). Most of the differential metabolites were involved in steroid hormone biosynthesis (Fig. 7B), vitamin digestion and absorption (Fig. 7C) and neuroactive ligand-receptor interaction (Fig. 7D).

### Correlation analysis of the fecal microbiota with metabolites in orlistat treated PCOS mice

**Fig. 8:**
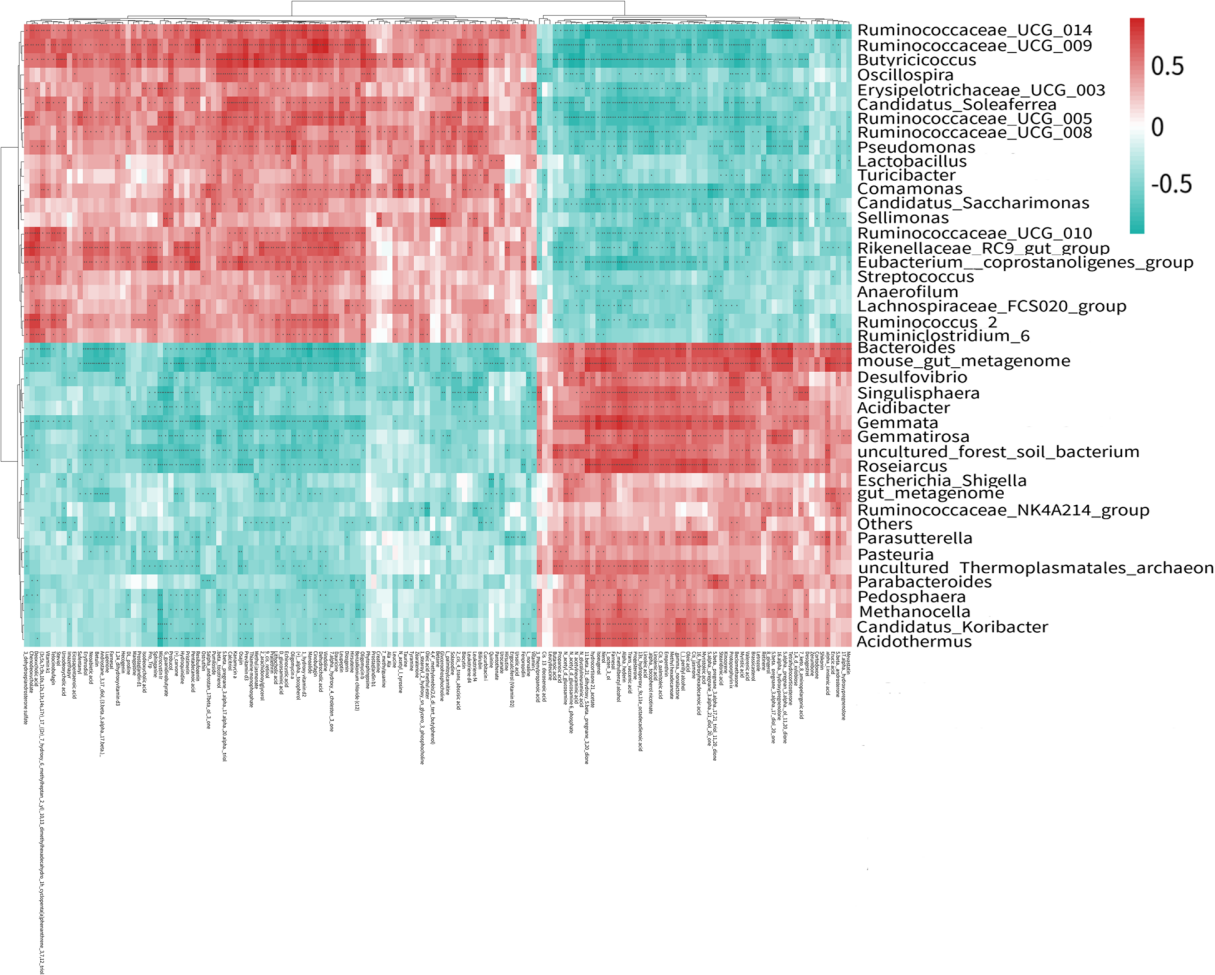
Correlation heatmap analysis of the differential fecal metabolites and gut microbiota between the PCOS and ORL-PCOS groups. A significant positive correlation is shown in red; a significant negative correlation is shown in blue, with darker colours indicating stronger correlations. * *p* < 0.05, ** *p* < 0.01, *** *p* < 0.001

The correlation between the differential metabolites and the microbial diversity in orlistat treated PCOS mice was analysed to further explore the composition and function of microorganisms. As shown in Fig. 8, we found that steroid hormone biosynthesis pathway related differential metabolites, such as 3-dehydroepiandrosterone sulfate (DHEAS), and 5alpha-androstan-17-beta-ol-3-one (dihydrotestosterone; DHT), were negatively correlated with the abundance of *Bacteroides*, and positively correlated with the abundances of *Ruminococcaceae_UCG_014*, *Ruminococcaceae_UCG_005* and *Pseudomonas.* Progesterone was positively correlated with the abundance of *Bacteroides*, and *Desulfovibrio* and negatively correlated with the abundance of *Ruminococcaceae_UCG_014* and *Ruminococcaceae_UCG_005* by correlation analysis.

## Discussion

Orlistat exerts an anti-obesity effect by inhibiting the absorption of triglycerides. Multiple clinical studies have proven its effect on weight loss [27, 28]. A recent study found that orlistat treatment has beneficial effects on body weight in HFD-induced obese mice by modifying the composition of the gut microbiota, presenting as reduced total microbial abundance and obviously upregulated bacteria [29]. In addition, 5% weight loss of initial body weight has been shown to increase ovulation frequency and fertility, and to improve testosterone and lipid levels in PCOS patients [30–32]. In this study, the gut microbiota and its metabolite profiles were first compared between PCOS mice and orlistat-treated PCOS mice, highlighting the critical role of microbiota and microbiota metabolites in controlling PCOS development, which could help to better understand the interaction between orlistat and PCOS.

Studies have shown that the gut microbiota is related to obesity or PCOS, and several phyla of microbiota may be involved in the occurrence and development of obesity or PCOS [14, 29, 33]. *Firmicutes* and *Bacteroidetes* are the two most abundant bacterial phyla in the human gut [34]. It has been found that the fecal community of obese mice/humans or PCOS patients/mouse models is characterized by an increased ratio of *Firmicutes* to *Bacteroidetes* (F/B) [35–37]. Changes in the F/B ratio were found during PCOS treatment. For example, Lin et al*.* observed a reduced abundance of *Firmicutes* and an elevated abundance of *Bacteroidetes* in response to sleeve gastrectomy in a dehydroepiandrosterone-induced PCOS rat model [38]. Additionally, flaxseed oil intervention modulated the gut microbiota and ameliorated PCOS in rats by decreasing the ratio of F/B [39]. A recent study found that orlistat could further decrease the F/B ratio in HFD-induced obese mice, exerting beneficial effects on body weight [29]. Consistently, in this study, we also found that the ORL-PCOS had significant changes in the composition of the gut microbiota, such as an increase in the abundance of *Bacteroidetes* and a decrease in *Firmicutes*. *Bacteroidetes* exert immunomodulatory effects on the host, and its elevation has been shown to be associated with weight loss in humans and animal models [40, 41]. Additionally, the *Bacteroides* genus belonging to the *Bacteroidetes* phylum was negatively associated with T2DM [42]. *Firmicutes*, which is closely related to obesity and metabolic syndrome, play a role in energy resorption and facilitate fat storage in the host body [11]. A previous study showed that orlistat is a reversible gastric and pancreatic lipase inhibitor and could promote weight loss by partially preventing intestinal fat absorption [43]. Weight loss is associated with changes in the gut microbiota [44]. Therefore, we speculated that orlistat may exert beneficial effects on body weight by modifying the gut microbiota, such as the decreased abundance of *Firmicutes,* which may help reduce fat storage and weight loss.

At the family level, we observed that orlistat treatment led to a significant decrease in the relative abundance of *Ruminococcaceae* and *Lactobacillaceae*, and an elevation of *Muribaculaceae* and *Bacteroidaceae*. Previous studies have found that the relative abundance of *Ruminococcaceae* was higher in obese patients with PCOS and letrozole-induced PCOS rat models [37, 45, 46]. Furthermore, the *Ruminococcaceae* abundance was reported to be associated with diabetes and testosterone levels. Mokkala et al. revealed that the relative abundance of *Ruminococcaceae* increased twofold in women with gestational diabetes compared to healthy controls [47]. The *Ruminococcus* genus can increase inflammatory cytokine production, which is positively associated with T2DM [42]. Eyupoglu et al. reported that *Ruminococcaceae* appears to be associated with clinical androgen excess in patients with PCOS [45]. *Lactobacillaceae* can ferment carbohydrates, including pectin and glucose, to produce formic acid, lactic acid, acetic acid and ethanol. Although *Lactobacillaceae* is commonly used as a probiotic, studies have shown that *Lactobacillaceae* is positively correlated with weight gain, and a higher level of *Lactobacillaceae* was detected in obese children and adults than in lean control individuals [48, 49], suggesting that the effect of *Lactobacillaceae* on metabolism may be species and strain specific [50]. Moreover, a significantly increased relative abundance of the *Lactobacillus* genus was observed in PCOS with insulin resistance rat models [46]. A lower level of *Lactobacillaceae* could alleviate obesity in HFD-induced obese mice. Ye et al. suggested that ripened pu-erh tea serves as a great candidate to alleviate obesity by increasing the abundance of *Bacteroidaceae*, *Muribaculaceae* and decreasing the abundance of *Lactobacillaceae* in HFD-induced obese mice [51], which is consistent with the results of the present study. *Bacteroidaceae* could promote cytokine production and have a potential role in inhibiting autoimmune disease [51]. Obese adolescents with PCOS had a lower relative abundance of the family *Bacteroidaceae*, which conferred a 4.4-fold higher likehood ratio of taxa predictive of PCOS diagnosis [52]. *Muribaculaceae* could degrade dietary components and polysaccharides to produce short chain fatty acids (SCFAs) that play key roles in anti-inflammatory and glycolipid homeostasis balance [53]. PCOS has been proven to be a chronic inflammatory disease, and obesity, insulin resistance, T2DM and hyperandrogenemia are common symptoms [26]. In the present study, we speculated that orlistat may improve the above PCOS symptoms by changing the gut microbiota composition, indicating that these bacteria might be the most efficient taxa contributing to preventing the development of PCOS.

Lipid metabolism is dysregulated in women with PCOS. Lipids are involved in various metabolic pathways, such as steroid hormone biosynthesis, and fatty acid metabolism [54]. In this study, based on the KEGG enrichment analysis, steroid hormone biosynthesis was the pathway in which the differential metabolites were most significantly enriched, such as DHEAS, dihydrotestosterone (DHT), progesterone and tetrahydrocorticosterone. The levels of DHEAS and DHT were significantly downregulated 0.40-fold and 0.16-fold respectively in orlistat treated PCOS mice compared with the control group. Correlation analysis revealed that both were negatively correlated with the abundance of *Bacteroides*, and positively correlated with the abundance of *Ruminococcaceae_UCG_014*, *Ruminococcaceae_UCG_005* and *Pseudomonas.* In a study on the fecal metabolites and gut microbiota in obese patients with PCOS, several fecal metabolites were used as characteristic metabolites, including DHEAS, which was significantly and positively correlated with the serum testosterone level and negatively correlated with body mass index (BMI) or fasting insulin among PCOS patients [26, 55]. DHEAS is a kind of androgen mainly secreted by the adrenal cortex, and found to be high in 22–25% of patients with PCOS [56]. Higher DHEAS could increase incidence of degenerated oocytes and early miscarriage rates in women with PCOS [57]. In addition, the prevalence of adrenal hyperandrogenaemia, which is defined as elevated circulating DHEAS levels, in women with PCOS is 15% to 45% [58]. Similar to DHEAS, the concentration of DHT, a potent androgen, was significantly higher in PCOS patients [59]. The DHT-induced PCOS model is one of the three typically used androgen-based PCOS models [60]. DHT exposure could cause the key reproductive characteristics (aperiodic, anovulatory and multifollicular ovaries) and metabolic characteristics (increased body weight and visceral adiposity) of PCOS [61]. Moreover, in this study, the level of progesterone was significantly upregulated by 6.74-fold in orlistat treated PCOS mice. Progesterone was positively correlated with the abundance of *Bacteroides*, and *Desulfovibrio* and negatively correlated with the abundance of *Ruminococcaceae_UCG_014* and *Ruminococcaceae_UCG_005* by correlation analysis. Progesterone is a key steroid hormone and is absolutely required for uterine implantation, decidualization, and maintenance of pregnancy [62]. PCOS women have lower progesterone levels during the luteal phase which overstimulates the immune system that produces more estrogen [63]. The chronic anovulation seen in PCOS implies long-term oestrogen excess or a lack of progesterone [64]. PCOS is a state of altered steroid hormone production and activity, and there is a close relationship between gut microbiota and sex hormones in PCOS [65]. Therefore, we speculated that orlistat may modify the gut microbiota of obese rats with PCOS, which has an impact on sex hormones, including decreased levels of DHEAS, and DHT and increased progesterone levels, to improve the steroid hormone state in PCOS.

## Conclusions

There are several limitations in this study. The number of samples was relatively small, a negative control group in the gut microbiome analysis was lacking, and the correlations were not verified. It is necessary to expand the sample size and verification in further research. This study was the first to use fecal microbiota combined with its metabolite profiles to explore the differences between PCOS mice and orlistat-treated PCOS mice, which may offer more specific mechanisms of orlistat treatment on PCOS and its related pathways. Based on 16S rRNA gene sequencing and untargeted metabolomics analysis, orlistat intervention modified the structure and composition of the gut microbiota, as well as the metabolite profiles of PCOS mice. Decreased abundance of *Firmicutes* and increased *Bacteroidetes* were observed in orlistat treated PCOS mice. Some families, such as *Ruminococcaceae* and *Lactobacillaceae,* which are associated with diabetes and testosterone levels or obesity, were decreased by orlistat intervention. *Muribaculaceae* and *Bacteroidaceae* which play anti-inflammatory roles, were notably enhanced by orlistat. The gut microbiota, fecal metabolites, and hormones were closely correlated. Steroid hormone biosynthesis was the pathway in which the differential metabolites were most significantly enriched.

## Supplementary Information

**Additional file 1.**

## Data Availability

The datasets presented in this study can be found in online repositories. The names of the repository/repositories and accession number(s) can be found below: NCBI SRA (accession: SUB12040976).

## References

[1] Teede HJ, Misso ML, Costello MF (2018). Recommendations from the international evidence-based guideline for the assessment and management of polycystic ovary syndrome. Hum Reprod.

[2] Rotterdam EA-SPcwg (2004). Revised 2003 consensus on diagnostic criteria and long-term health risks related to polycystic ovary syndrome (PCOS). Hum Reprod.

[3] Li R, Zhang Q, Yang D (2013). Prevalence of polycystic ovary syndrome in women in China: a large community-based study. Hum Reprod.

[4] Azziz R, Carmina E, Chen Z (2016). Polycystic ovary syndrome. Nat Rev Dis Primers.

[5] Li T, Wu K, You L (2013). Common variant rs9939609 in gene FTO confers risk to polycystic ovary syndrome. PLoS one.

[6] Giampaolino P, Foreste V, Di Filippo C (2021). Microbiome and PCOS: State-of-Art and Future Aspects. Int J Mol Sci.

[7] Qi X, Yun C, Sun L (2019). Gut microbiota-bile acid-interleukin-22 axis orchestrates polycystic ovary syndrome. Nat Med.

[8] Xu WL, Liu GY, Zhang N (2023). Untargeted metabolomics analysis of serum and follicular fluid samples from women with polycystic ovary syndrome. Minerva Endocrinol..

[9] Barko PC, McMichael MA, Swanson KS (2018). The gastrointestinal microbiome: a review. J Vet Intern Med.

[10] Cani PD, Amar J, Iglesias MA (2007). Metabolic endotoxemia initiates obesity and insulin resistance. Diabetes.

[11] Turnbaugh PJ, Ley RE, Mahowald MA (2006). An obesity-associated gut microbiome with increased capacity for energy harvest. Nature.

[12] Torres PJ, Siakowska M, Banaszewska B (2018). Gut microbial diversity in women with polycystic ovary syndrome correlates with hyperandrogenism. J Clin Endocrinol Metab.

[13] Sun L, Hu W, Liu Q (2012). Metabonomics reveals plasma metabolic changes and inflammatory marker in polycystic ovary syndrome patients. J Proteome Res.

[14] Zhao X, Jiang Y, Xi H (2020). Exploration of the Relationship Between Gut Microbiota and Polycystic Ovary Syndrome (PCOS): a Review. Geburtshilfe Frauenheilkd.

[15] Shi Y, Guo M, Yan J (2007). Analysis of clinical characteristics in large-scale Chinese women with polycystic ovary syndrome. Neuro Endocrinol Lett.

[16] Dahan MH, Reaven G (2019). Relationship among obesity, insulin resistance, and hyperinsulinemia in the polycystic ovary syndrome. Endocrine.

[17] Brassard M, AinMelk Y, Baillargeon JP (2008). Basic infertility including polycystic ovary syndrome. Med Clin North Am.

[18] Lim SS, Norman RJ, Davies MJ (2013). The effect of obesity on polycystic ovary syndrome: a systematic review and meta-analysis. Obes Rev.

[19] Moran LJ, Pasquali R, Teede HJ (2009). Treatment of obesity in polycystic ovary syndrome: a position statement of the Androgen Excess and Polycystic Ovary Syndrome Society. Fertil Steril.

[20] Li Y, Chen C, Ma Y (2019). Multi-system reproductive metabolic disorder: significance for the pathogenesis and therapy of polycystic ovary syndrome (PCOS). Life Sci.

[21] Kumar P, Arora S (2014). Orlistat in polycystic ovarian syndrome reduces weight with improvement in lipid profile and pregnancy rates. J Hum Reprod Sci.

[22] Panidis D, Farmakiotis D, Rousso D (2008). Obesity, weight loss, and the polycystic ovary syndrome: effect of treatment with diet and orlistat for 24 weeks on insulin resistance and androgen levels. Fertil Steril.

[23] Wu YX, Yang XY, Han BS (2022). Naringenin regulates gut microbiota and SIRT1/ PGC-1a signaling pathway in rats with letrozole-induced polycystic ovary syndrome. Biomed Pharmacother.

[24] Lucas N, Legrand R, Deroissart C (2019). Hafnia alvei HA4597 strain reduces food intake and body weight gain and improves body composition, glucose, and lipid metabolism in a mouse model of hyperphagic obesity. Microorganisms.

[25] Peng MF, Tian S, Song YG (2021). Effects of total flavonoids from Eucommia ulmoides Oliv. leaves on polycystic ovary syndrome with insulin resistance model rats induced by letrozole combined with a high-fat diet. J Ethnopharmacol.

[26] Zhou L, Ni Z, Yu J (2020). Correlation between fecal metabolomics and gut microbiota in obesity and polycystic ovary syndrome. Front Endocrinol (Lausanne).

[27] Sahebkar A, Simental-Mendia LE, Reiner Z (2017). Effect of orlistat on plasma lipids and body weight: a systematic review and meta-analysis of 33 randomized controlled trials. Pharmacol Res.

[28] Aldekhail NM, Logue J, McLoone P (2015). Effect of orlistat on glycaemic control in overweight and obese patients with type 2 diabetes mellitus: a systematic review and meta-analysis of randomized controlled trials. Obes Rev.

[29] Ke J, An Y, Cao B (2020). Orlistat-induced gut microbiota modification in obese mice. Evid Based Complement Alternat Med.

[30] Legro RS (2012). Obesity and PCOS: implications for diagnosis and treatment. Semin Reprod Med.

[31] Pasquali R, Gambineri A, Pagotto U (2006). The impact of obesity on reproduction in women with polycystic ovary syndrome. BJOG.

[32] Motta AB (2012). The role of obesity in the development of polycystic ovary syndrome. Curr Pharm Des.

[33] Wang P, Gao J, Ke W (2020). Resveratrol reduces obesity in high-fat diet-fed mice via modulating the composition and metabolic function of the gut microbiota. Free Radic Biol Med.

[34] John GK, Mullin GE (2016). The gut microbiome and obesity. Curr Oncol Rep.

[35] Gomes AC, Hoffmann C, Mota JF (2018). The human gut microbiota: Metabolism and perspective in obesity. Gut Microbes.

[36] Lindheim L, Bashir M, Munzker J (2017). Alterations in gut microbiome composition and barrier function are associated with reproductive and metabolic defects in women with Polycystic Ovary Syndrome (PCOS): a pilot study. PLoS one.

[37] Kelley ST, Skarra DV, Rivera AJ (2016). The gut microbiome is altered in a letrozole-induced mouse model of polycystic ovary syndrome. PLoS one.

[38] Lin W, Wen L, Wen J (2021). Effects of sleeve gastrectomy on fecal gut microbiota and short-chain fatty acid content in a rat model of polycystic ovary syndrome. Front Endocrinol (Lausanne).

[39] Wang T, Sha L, Li Y (2020). Dietary alpha-Linolenic acid-rich flaxseed oil exerts beneficial effects on polycystic ovary syndrome through sex steroid hormones-microbiota-inflammation axis in rats. Front Endocrinol (Lausanne).

[40] Liu R, Hong J, Xu X (2017). Gut microbiome and serum metabolome alterations in obesity and after weight-loss intervention. Nat Med.

[41] Turnbaugh PJ, Hamady M, Yatsunenko T (2009). A core gut microbiome in obese and lean twins. Nature.

[42] Gurung M, Li Z, You H (2020). Role of gut microbiota in type 2 diabetes pathophysiology. EBioMedicine.

[43] Padwal RS, Majumdar SR (2007). Drug treatments for obesity: orlistat, sibutramine, and rimonabant. Lancet.

[44] Koutoukidis DA, Jebb SA, Zimmerman M (2022). The association of weight loss with changes in the gut microbiota diversity, composition, and intestinal permeability: a systematic review and meta-analysis. Gut Microbes.

[45] Eyupoglu ND, Ergunay K, Acikgoz A (2020). Gut microbiota and oral contraceptive use in overweight and obese patients with polycystic ovary syndrome. J Clin Endocrinol Metab.

[46] Zhu Y, Li Y, Liu M (2020). Guizhi Fuling Wan, Chinese Herbal Medicine, Ameliorates Insulin Sensitivity in PCOS Model Rats With Insulin Resistance via Remodeling Intestinal Homeostasis. Front Endocrinol (Lausanne).

[47] Mokkala K, Houttu N, Vahlberg T (2017). Gut microbiota aberrations precede diagnosis of gestational diabetes mellitus. Acta Diabetol.

[48] Jin J, Cheng R, Ren Y (2021). Distinctive gut microbiota in patients with overweight and obesity with dyslipidemia and its responses to long-term orlistat and ezetimibe intervention: a randomized controlled open-label trial. Front Pharmacol.

[49] Bervoets L, Van Hoorenbeeck K, Kortleven I (2013). Differences in gut microbiota composition between obese and lean children: a cross-sectional study. Gut Pathog.

[50] Million M, Angelakis E, Paul M (2012). Comparative meta-analysis of the effect of Lactobacillus species on weight gain in humans and animals. Microb Pathog.

[51] Ye J, Zhao Y, Chen X (2021). Pu-erh tea ameliorates obesity and modulates gut microbiota in high fat diet fed mice. Food Res Int.

[52] Jobira B, Frank DN, Pyle L (2020). Obese adolescents with PCOS have altered biodiversity and relative abundance in gastrointestinal microbiota. J Clin Endocrinol Metab.

[53] Li LL, Wang YT, Zhu LM (2020). Inulin with different degrees of polymerization protects against diet-induced endotoxemia and inflammation in association with gut microbiota regulation in mice. Sci Rep.

[54] Rajska A, Buszewska-Forajta M, Rachon D (2020). Metabolomic insight into polycystic ovary syndrome-an overview. Int J Mol Sci.

[55] Moran C, Arriaga M, Arechavaleta-Velasco F (2015). Adrenal androgen excess and body mass index in polycystic ovary syndrome. J Clin Endocrinol Metab.

[56] Khan SH, Rizvi SA, Shahid R (2021). Dehydroepiandrosterone Sulfate (DHEAS) Levels in Polycystic Ovarian Syndrome (PCOS). J Coll Physicians Surg Pak.

[57] Jimenez PT, Frolova AI, Chi MM (2013). DHEA-mediated inhibition of the pentose phosphate pathway alters oocyte lipid metabolism in mice. Endocrinology.

[58] Luque-Ramirez M, Escobar-Morreale HF (2016). Adrenal hyperandrogenism and polycystic ovary syndrome. Curr Pharm Des.

[59] O'Reilly MW, Kempegowda P, Walsh M (2017). AKR1C3-mediated adipose androgen generation drives lipotoxicity in women with polycystic ovary syndrome. J Clin Endocrinol Metab.

[60] Walters KA, Allan CM, Handelsman DJ (2012). Rodent models for human polycystic ovary syndrome. Biol Reprod.

[61] Rodriguez Paris V, Edwards MC, Aflatounian A (2021). Pathogenesis of reproductive and metabolic PCOS traits in a mouse model. J Endocr Soc.

[62] Wetendorf M, DeMayo FJ (2012). The progesterone receptor regulates implantation, decidualization, and glandular development via a complex paracrine signaling network. Mol Cell Endocrinol.

[63] Luan YY, Zhang L, Peng YQ (2022). Immune regulation in polycystic ovary syndrome. Clin Chim Acta.

[64] Li X, Feng Y, Lin JF (2014). Endometrial progesterone resistance and PCOS. J Biomed Sci.

[65] Insenser M, Murri M, Del Campo R (2018). Gut microbiota and the polycystic ovary syndrome: influence of sex, sex hormones, and obesity. J Clin Endocrinol Metab.

